# Temporal Trends in the Use of Regional Anesthesia for Hip Fracture Surgery: A Retrospective Single-Center Study

**DOI:** 10.7759/cureus.86636

**Published:** 2025-06-24

**Authors:** Casey J Kukielski, Brittany L Deiling, Bhiken I Naik, Brian J Reon, Seth R Yarboro, Nabil Elkassabany

**Affiliations:** 1 Anesthesiology, Wellstar Kennestone Hospital, Marietta, USA; 2 Anesthesiology, University of Virginia Health System, Charlottesville, USA; 3 Orthopedic Surgery, University of Virginia Health System, Charlottesville, USA

**Keywords:** general anesthesia, hip fracture surgery, neuraxial anesthesia, peripheral nerve block, regional anesthesia

## Abstract

Introduction: Hip fractures are associated with significant morbidity and mortality in the United States. Over the past decade, considerable research has been directed toward identifying the optimal anesthetic and analgesic techniques for hip fracture surgery. It was hypothesized that regional anesthesia (neuraxial anesthesia and peripheral nerve blocks) may improve outcomes by avoiding the risks of general anesthesia and providing opioid-sparing analgesia in these complex patients. For neuraxial anesthesia, evidence of clinical benefit has been inconsistent in the literature; however, studies on peripheral nerve block analgesia have shown more consistent advantages. It is unclear if this data has influenced changes in clinical practice.

Materials and Methods: We conducted a retrospective single-center study of patients who underwent hip fracture surgery at the University of Virginia Medical Center from 2018 to 2022. Descriptive statistics were produced for patient demographics and clinical characteristics, and generalized linear regression models were used to examine trends in anesthetic techniques (general versus neuraxial anesthesia and use of peripheral nerve blocks) over time. We also examined the association between regional anesthesia and patient outcomes, including hospital length of stay (primary outcome), postoperative pain scores, incidence of acute kidney injury and myocardial injury, and discharge disposition (secondary outcomes), as well as diurnal variation in regional anesthesia utilization.

Results: We observed a 30% increase in the number of neuraxial anesthesia cases per year (from 10% (n = 12) of patients in 2018 to 33% (n = 40) in 2022), as well as an increase in peripheral nerve block utilization over time (1.7% (n = 2) of patients in 2018 to 28.1% (n = 34) in 2022). Neuraxial anesthesia was associated with a 17.1% lower hospital length of stay compared to general anesthesia (primary outcome, mean 6.2 vs 7.4 days, p = 0.006). There were no other differences in postoperative outcomes, and there was no evidence of diurnal variation in regional anesthesia utilization.

Conclusions: The increase in peripheral nerve block utilization demonstrates continued progress in quality of care, while emphasizing that there is still significant room for improvement. A larger multicenter database study will be helpful to assess if the evolving literature has influenced broader changes in clinical practice.

## Introduction

Hip fractures are associated with significant morbidity and an estimated one-year mortality of 21% in the United States [1-3]. There are roughly 300,000 admissions for hip fractures per year in the United States, and it is anticipated that the incidence will triple by 2050 in the setting of an aging population [4,5]. As part of multidisciplinary efforts to improve patient outcomes, considerable research has been directed toward identifying the optimal anesthetic and analgesic techniques for hip fracture surgery over the last decade.

It was hypothesized that regional anesthesia (neuraxial anesthesia and peripheral nerve blocks) may improve outcomes by avoiding the risks of general anesthesia and providing opioid-sparing analgesia in these complex patients. For neuraxial anesthesia, evidence of clinical benefit has been inconsistent in the literature. Initial observational studies reported that neuraxial anesthesia was associated with decreased pulmonary complications [6-8], hospital length of stay [7-9], and mortality [6-8,10] compared to general anesthesia. However, the REGAIN and RAGA trials, two large prospective randomized multicenter studies published in 2021 and 2022, concluded that neuraxial anesthesia was not superior to general anesthesia with respect to postoperative delirium, pain scores, hospital length of stay, inability to walk independently at 60 days, or overall survival [11-13]. In contrast, the literature on peripheral nerve block analgesia has yielded more consistent benefits. A 2020 Cochrane review of 49 randomized controlled trials reported high-certainty evidence that peripheral nerve blocks for hip fractures reduce pain on movement and risk of delirium, with moderate-certainty evidence supporting reduced risk of chest infection and time to first mobilization [14]. In conclusion, the authors stated that “some trials are ongoing, but it is unclear whether any further randomized controlled trials (RCTs) should be registered, given the benefits found.”

Prior to the REGAIN and RAGA trials, analysis of the National Surgical Quality Improvement Program (NSQIP) database revealed a roughly 50% increase (from 15.1% to 22.9%) in the use of spinal anesthesia for hip fracture surgery from 2007 to 2017, corresponding with the growth of an elderly subpopulation [15]. A review of a large U.S. private insurer database noted that although the use of peripheral nerve blocks for hip fracture analgesia increased from 2004 to 2016, utilization remained below five percent despite guidelines recommending routine use [16-18]. However, there have not been any follow-up studies to assess more recent trends in anesthetic and analgesic techniques for hip fracture surgery, so it remains unclear if the evolving literature has influenced changes in clinical practice.

Our study aims to investigate the temporal trends in anesthesia techniques (general versus neuraxial anesthesia and use of peripheral nerve blocks for analgesia) for hip fracture repair at our academic institution over the last five years. We hypothesize that the proportion of cases receiving neuraxial anesthesia and peripheral nerve blocks has increased over time. We also examined the association of anesthetic technique with postoperative outcomes, including hospital length of stay (primary outcome), postoperative pain scores (through postoperative day three), incidence of acute kidney injury and myocardial injury, and discharge disposition (secondary outcomes).

An additional exploratory aim was to assess potential diurnal variation in regional anesthesia and analgesia utilization. At our institution, fellowship-trained regional anesthesia faculty are reliably available to perform neuraxial anesthesia and peripheral nerve blocks for patients undergoing hip fracture surgery during the day; however, the on-call anesthesiologist may not perform these procedures regularly or have the time or resources to do the procedures overnight. We hypothesize that patients operated on outside of normal business hours (after 5 pm) are less likely to receive regional anesthesia. If diurnal variation in regional anesthesia utilization does exist, it is important to recognize it, so we can improve scheduling practices or adapt perioperative workflow to ensure all patients receive the best care possible, regardless of the time of surgery.

## Materials and methods

We conducted a retrospective single-center study of all adult patients who underwent hip fracture surgery at the University of Virginia (UVA) Medical Center from January 9, 2018, to December 3, 2022. Data was obtained from our departmental database, which stores de-identified perioperative data from the Epic electronic medical record (EMR). We included all patients who underwent hip fracture surgery during the study period, and no cases were excluded. Patients were identified by Current Procedural Terminology (CPT) codes for hip fracture surgery and data pertaining to patient demographics (such as age, sex, and race), clinical characteristics (such as BMI, Elixhauser score, time from admission to surgery, surgery date and time, anesthesia type, peripheral nerve block utilization, oral morphine equivalent (OME) opioid requirement, transfusion requirements, and intraoperative hypotension), and postoperative outcomes (such as hospital length of stay, postoperative pain scores (through POD 3), incidence of acute kidney injury and myocardial injury, and discharge disposition) were reviewed systematically. Patients who received spinal, epidural, or combined spinal-epidural anesthesia were categorized as neuraxial anesthesia, and patients who received fascia iliaca, femoral, sciatic, pericapsular nerve group (PENG), or lateral femoral cutaneous nerve (LFCN) blocks were included in the peripheral nerve block group. Cases with missing data were excluded from the analysis of corresponding outcomes.

Statistical analysis was performed using R version 4.3.0 (R Foundation for Statistical Computing, Vienna, Austria) with the lme4 package for generalized linear regression models. Descriptive statistics were presented as means, standard deviations, medians and interquartile ranges (for continuous variables), or frequencies and proportions (for categorical variables). Generalized linear regression models with a Poisson link were used to examine trends in anesthetic techniques over time, as well as the association between regional anesthesia and patient outcomes. We selected this approach as the dependent variables of interest are count variables, and there was no evidence of over-dispersion in our dataset. We included age, sex, race, BMI, Elixhauser score, year, time from admission to surgery, surgery time of day, intraoperative OME (mg), intraoperative blood transfusion (number of units), area under the curve (AUC) for mean arterial blood pressure (MAP) < 60 mmHg (mmHg*minutes), and surgery duration (hours) as covariates in the model. To allow for variables of similar scale, age is divided by 10, surgery duration was operationalized as hours, and time from admission to surgery was operationalized in days. We controlled for the included covariates in our analysis of the primary outcome of length of stay, and no variables were shown to be collinear. In our investigation of diurnal variation in regional anesthesia utilization, we chose 5 pm as the cutoff for daytime versus overnight surgery since that is the start time for the on-call (overnight) anesthesiologist at our institution.

This study was determined to be exempt from the University of Virginia Institutional Review Board for Health Sciences Research (UVA IRB-HSR) review. Pertinent data were accessed by our departmental data scientist on January 27, 2023. The authors did not have access to protected health information during the study: A de-identified dataset was received for statistical analysis, and the data were analyzed anonymously.

## Results

We identified 620 patients who underwent hip fracture repair at our institution over five years, with a relatively homogenous distribution of cases over time (range: 117-132 cases per year). Most patients were females (n = 394, 63.5%). The majority of patients self-identified as white (n = 570, 92.1%), with smaller proportions as black (n = 31, 5%) and other (n = 18, 2.9%). Additional patient demographic and clinical characteristics are detailed in Table 1. Most cases (n = 523, 84.3%) started before 5 pm.

**Table 1:** Descriptive statistics of patient demographic and clinical characteristics BMI: Body mass index; OME: Oral morphine equivalent; AUC: Area under the curve; MAP: Mean arterial pressure; h: hours; min: minutes; mg: milligrams.

	n	M	SD	25^th^-ile	Median	75^th^-ile
Age	620	74.31	10.99	66.0	7	84
BMI	603	26.28	6.51	21.9	25	29
Elixhauser score	458	3.06	6.29	-1.0	0	6
Time from admission to surgery (h)	620	31.04	42.90	16.0	22	31
Surgery duration (min)	620	95.01	50.96	68.0	85	107
Intra-op OME (mg)	412	17.04	17.55	7.5	12	21
Intra-op transfusion (#)	620	0.08	0.34	0.0	0	0
AUC for MAP < 60mmHg (mmHg*minutes)	620	367.32	523.80	0.0	200	546
Post-op OME (mg)	118	31.38	97.00	7.5	15	21

**Figure 1:**
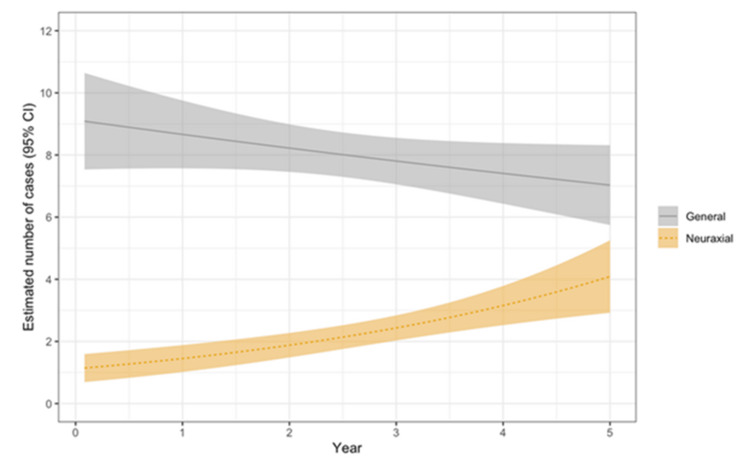
Estimated number of general and neuraxial anesthesia cases over time. Shaded areas depict 95% confidence intervals. Fitted line graph of generalized linear regression model.

In total, 481 patients (77.6%) received general anesthesia, and 139 patients (22.4%) received neuraxial (spinal or epidural) anesthesia. Descriptive statistics of the frequency and proportion of cases by anesthesia group per year are detailed in Table 2. Estimated trends in general versus neuraxial anesthesia are depicted in Figure 1. Results from a generalized linear regression model showed a 30% increase in the number of neuraxial anesthesia cases per year (from 10% (n = 12) of patients in 2018 to 33% (n = 40) in 2022), whereas there was no statistically significant change in the number of general anesthesia cases over time.

**Table 2:** Frequency and proportion of cases by anesthesia group per year

	General		Neuraxial	
Year	n	%	n	%
2018	108	90	12	10
2019	99	85	18	15
2020	96	73	36	27
2021	97	75	33	25
2022	81	67	40	33

Statistical results of patient outcomes by anesthesia group are summarized in Table 3. Neuraxial anesthesia was associated with a 17.1% lower hospital length of stay compared to general anesthesia (primary outcome, mean 6.2 vs 7.4 days, incidence rate ratio (IRR) = 0.83, 95% confidence interval (CI): 0.72-0.95, p = 0.006). There were no significant differences in the postoperative pain scores (95% CI: -0.77-0.45, p = 0.601), incidence of acute kidney injury (95% CI: -0.33-0.12, p = 0.373), or odds of being discharged home (95% CI: 0.15-2.40, p = 0.599) between the two groups, and there was insufficient data to perform meaningful statistical analysis regarding postoperative myocardial injury (secondary outcomes). There was no difference in the proportion of patients who received general versus neuraxial anesthesia in cases starting before and after 5 pm (p = 0.466).

**Table 3:** Statistical results of patient outcomes by anesthesia group IRR: Incidence rate ratio; CI: Confidence interval.

Neuraxial vs general anesthesia			
	IRR	95% CI	p-value
Length of stay (days)	0.83	0.72–0.95	0.006
Postoperative pain scores		-0.77–0.45	0.601
Acute kidney injury		-0.33–0.12	0.373
Discharge home		0.15–2.40	0.599
Peripheral nerve block vs no nerve block			
	IRR	95% CI	p-value
Length of stay (days)	1.13	0.97–1.31	0.105
Postoperative pain scores		-0.63–1.01	0.647
Acute kidney injury		-0.27–0.34	0.819
Discharge home		0.14–4.40	0.988

**Figure 2:**
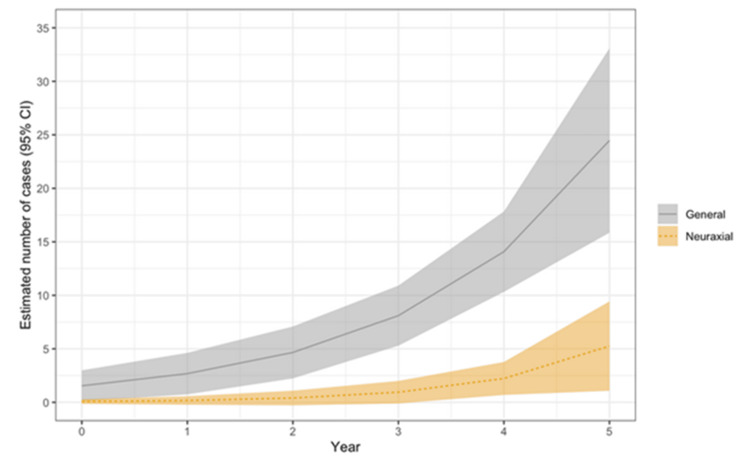
Estimated number of general and neuraxial anesthesia cases that received peripheral nerve blocks over time. Shaded areas depict 95% confidence intervals. Fitted line graph of generalized linear regression model.

Sixty-three patients (10.16%) received peripheral nerve blocks for postoperative analgesia; the majority received fascia iliaca blocks, with a lower percentage of femoral nerve blocks and a small number of PENG blocks. Estimated trends in the use of peripheral nerve blocks are depicted in Figure 2. Cumulatively, peripheral nerve block utilization increased from 1.7% (n = 2) of patients in 2018 to 28.1% (n = 34) in 2022. The results showed a statistically significant increase in the number of general anesthesia cases that received nerve blocks over time, whereas the number of neuraxial cases that received nerve blocks remained low. There were no significant differences in the hospital length of stay (primary outcome, IRR = 1.13, 95% CI: 0.97-1.31, p = 0.105), postoperative pain scores (95% CI: -0.63-1.01, p = 0.647), incidence of acute kidney injury (95% CI: -0.27-0.34, p = 0.819), or odds of being discharged home (95% CI: 0.14-4.40, p = 0.988) between the two groups, and there was insufficient data to perform meaningful statistical analysis regarding postoperative myocardial injury (secondary outcomes). There was no difference in the proportion of patients who received nerve blocks in surgeries starting before and after 5 pm (p = 0.896).

## Discussion

Our analysis revealed trends of increasing use of neuraxial anesthesia and peripheral nerve blocks for hip fracture surgery at our institution over time, and neuraxial anesthesia was associated with lower hospital length of stay. There were no other differences in postoperative outcomes (such as average pain scores through POD 3, incidence of acute kidney injury, or discharge disposition) between patients who received general versus neuraxial anesthesia or peripheral nerve block versus no nerve block, and there was no evidence of diurnal variation in neuraxial anesthesia or peripheral nerve block utilization.

The trend of increasing use of neuraxial anesthesia is consistent with a prior observational study, which reported a 50% increase in the use of spinal anesthesia for hip fracture surgery from 2007 to 2017, corresponding with the growth of an elderly subpopulation [15]. Unfortunately, the proposed benefits of neuraxial anesthesia have not yielded reproducible outcome improvements in the literature. Our finding that neuraxial anesthesia was associated with lower hospital length of stay is in line with prior retrospective studies that reported decreased pulmonary complications [6-8], hospital length of stay [7-9], and mortality [6-8,10] compared to general anesthesia; however, these benefits were not observed in subsequent prospective trials. The REGAIN trial showed similar risk of delirium, time from randomization to discharge, inability to walk independently at 60 days, and 60-day mortality in patients who received spinal or general anesthesia; additionally, secondary analysis revealed that spinal anesthesia was associated with more pain in the first 24 hours and higher prescription analgesic use at 60 days [11,13]. This was corroborated by the RAGA trial, which reported similar incidence of postoperative delirium, pain scores, length of hospitalization, and 30-day mortality in patients who received neuraxial or general anesthesia [12]. Considering the lack of benefits observed in these prospective trials, we believe the association between neuraxial anesthesia and lower hospital length of stay is more likely related to confounding from unmeasured variables such as preoperative functional status or medical comorbidities than true causality. Given the current literature, we believe there is insufficient evidence to support preferential use of neuraxial anesthesia for hip fracture surgery, and the decision between neuraxial and general anesthesia should largely depend on individual practice style and patient-specific factors.

Fortunately, studies on peripheral nerve blocks for hip fracture analgesia have yielded more consistent benefits. Cumulatively, a 2020 Cochrane review concluded there is high-certainty evidence of reduced pain on movement and risk of delirium, as well as moderate-certainty evidence supporting reduced risk of chest infection and time to first mobilization [14]. However, prior observational studies noted persistently low utilization despite guidelines recommending routine use [16-18]. We were pleased with the trend of increasing peripheral nerve block utilization at our institution over time. However, there remains significant room for improvement, as only 28.1% (n = 34) of hip fracture patients received nerve blocks in the last year of the study. We were surprised that the increase in peripheral nerve blocks was mainly in the general anesthesia group, whereas the number of neuraxial cases that received nerve blocks remained low. While patients who receive spinal or epidural anesthesia may have lower analgesic requirements until the neuraxial blockade resolves, it is important to note that all hip fracture patients may benefit from peripheral nerve blocks for opioid-sparing analgesia regardless of intraoperative anesthesia technique [17-18]. The lack of difference in postoperative pain scores in patients who received peripheral nerve blocks was also unexpected; it is possible that this absence of effect is due to our lack of differentiation between static and dynamic pain scores or confounding from increased opioid administration in patients who did not receive nerve blocks. However, this is outweighed by higher level evidence and multispecialty guidelines with strong recommendations for routine use [14,17-20].

In our investigation of potential diurnal variation in regional anesthesia utilization, we were surprised by the lack of difference in the proportion of patients who received peripheral nerve blocks in surgeries starting before and after 5 pm. We would have anticipated increased regional anesthesia utilization during regular working hours at a tertiary academic center with a dedicated regional anesthesia division. The lack of difference in spinal anesthesia utilization may be attributed to most general anesthesiologists being comfortable performing neuraxial anesthesia, and the low proportions of patients who received peripheral nerve blocks and cases starting after 5 pm may have limited the power to detect diurnal variation in peripheral nerve block utilization. As such, future studies with a larger cohort could help substantiate the presence or absence of a time-of-day effect.

Our study has numerous limitations stemming from its retrospective single-center design and relatively small cohort. Retrospective studies are susceptible to both selection and information bias, which inherently limit generalizability. In this study, missing covariate data (such as BMI, Elixhauser score, intraoperative, and postoperative OME) and unmeasured variables (such as preoperative functional status, medical comorbidities, and variability in postoperative analgesic management) are sources of potential information bias and confounding, which may reduce statistical power and result in biased estimates. As previously discussed, our small cohort also limits the power to detect smaller effect sizes and increases the risk of type II errors.

Despite these limitations, we believe this study demonstrates ongoing progress in quality of care pertaining to peripheral nerve block utilization for hip fracture analgesia at our institution, while emphasizing that there is still significant room for improvement. Following publication of our single-center data, we plan to proceed with a larger database study to examine broader trends in regional anesthesia utilization for hip fracture surgery across the United States.

## Conclusions

Our study revealed trends of increasing use of neuraxial anesthesia and peripheral nerve blocks for hip fracture surgery at our institution over time, and there was no evidence of diurnal variation in regional anesthesia utilization. Notably, the increase in peripheral nerve block utilization demonstrates continued progress in quality of care, while emphasizing that there is still significant room for improvement. A larger multicenter database study will be helpful to assess if the evolving literature has influenced broader changes in clinical practice.
